# Supplementary material for: A UMLS-based spell checker for natural language processing in vaccine safety
Source: BMC Med Inform Decis Mak. 2007 Feb 12;7:3. doi: 10.1186/1472-6947-7-3 (PMC1805499; doi:10.1186/1472-6947-7-3)
Supplement: Additional file 13 — RAP application directory. Sets up directory for RAP (RDF application for PHP) source code [file 1472-6947-7-3-S13.gz › rap/doc/phpdoc/__filesource/fsource_default__vocabularyFOAF_RES.php.html]

xml version="1.0" encoding="iso-8859-1"?


File Source for FOAF\_RES.php


# Source for file FOAF\_RES.php

Documentation is available at FOAF\_RES.php

```
1. <?PHP
2. // ----------------------------------------------------------------------------------
3. // Friend of a Friend (FOAF) Vocabulary (ResResource)
4. // ----------------------------------------------------------------------------------
5. // Version                   : 0.9
6. // Authors                   : Tobias Gau� (tobias.gauss@web.de)
7. //
8. // Description               : Wrapper, defining resources for all terms of the
9. //                               Friend of a Friend project (FOAF).
10. //                               For details about FOAF see: http://xmlns.com/foaf/0.1/.
11. //                                Using the wrapper allows you to define all aspects of
12. //                             the vocabulary in one spot, simplifing implementation and
13. //                             maintainence.
14. //
15. // ----------------------------------------------------------------------------------
16. // History:
17. // 11-08-2003                 : Initial version
18. // ----------------------------------------------------------------------------------


26. class FOAF_RES{

28. function AGENT()
29. {
30. return  new ResResource(FOAF_NS . 'Agent');

32. }

34. function DOCUMENT()
35. {
36. return  new ResResource(FOAF_NS . 'Document');

38. }

40. function GROUP()
41. {
42. return  new ResResource(FOAF_NS . 'Group');

44. }

46. function IMAGE()
47. {
48. return  new ResResource(FOAF_NS . 'Image');

50. }

52. function ONLINE_ACCOUNT()
53. {
54. return  new ResResource(FOAF_NS . 'OnlineAccount');

56. }

58. function ONLINE_CHAT_ACCOUNT()
59. {
60. return  new ResResource(FOAF_NS . 'OnlineChatAccount');

62. }

64. function ONLINE_ECOMMERCE_ACCOUNT()
65. {
66. return  new ResResource(FOAF_NS . 'OnlineEcommerceAccount');

68. }

70. function ONLINE_GAMING_ACCOUNT()
71. {
72. return  new ResResource(FOAF_NS . 'OnlineGamingAccount');

74. }

76. function ORGANIZATION()
77. {
78. return  new ResResource(FOAF_NS . 'Organization');

80. }

82. function PERSON()
83. {
84. return  new ResResource(FOAF_NS . 'Person');

86. }

88. function PERSONAL_PROFILE_DOCUMENT()
89. {
90. return  new ResResource(FOAF_NS . 'PersonalProfileDocument');

92. }

94. function PROJECT()
95. {
96. return  new ResResource(FOAF_NS . 'Project');

98. }

100. function ACCOUNT_NAME()
101. {
102. return  new ResResource(FOAF_NS . 'accountName');

104. }

106. function ACCOUNT_SERVICE_HOMEPAGE()
107. {
108. return  new ResResource(FOAF_NS . 'accountServiceHomepage');

110. }

112. function AIM_CHAT_ID()
113. {
114. return  new ResResource(FOAF_NS . 'aimChatID');

116. }

118. function BASED_NEAR()
119. {
120. return  new ResResource(FOAF_NS . 'based_near');

122. }

124. function CURRENT_PROJECT()
125. {
126. return  new ResResource(FOAF_NS . 'currentProject');

128. }

130. function DEPICTION()
131. {
132. return  new ResResource(FOAF_NS . 'depiction');

134. }

136. function DEPICTS()
137. {
138. return  new ResResource(FOAF_NS . 'depicts');

140. }

142. function DNA_CHECKSUM()
143. {
144. return  new ResResource(FOAF_NS . 'dnaChecksum');

146. }

148. function FAMILY_NAME()
149. {
150. return  new ResResource(FOAF_NS . 'family_name');

152. }

154. function FIRST_NAME()
155. {
156. return  new ResResource(FOAF_NS . 'firstName');

158. }

160. function FUNDED_BY()
161. {
162. return  new ResResource(FOAF_NS . 'fundedBy');

164. }

166. function GEEKCODE()
167. {
168. return  new ResResource(FOAF_NS . 'geekcode');

170. }

172. function GENDER()
173. {
174. return  new ResResource(FOAF_NS . 'gender');

176. }

178. function GIVENNAME()
179. {
180. return  new ResResource(FOAF_NS . 'givenname');

182. }

184. function HOLDS_ACCOUNT()
185. {
186. return  new ResResource(FOAF_NS . 'holdsAccount');

188. }

190. function HOMEPAGE()
191. {
192. return  new ResResource(FOAF_NS . 'homepage');

194. }

196. function ICQ_CHAT_ID()
197. {
198. return  new ResResource(FOAF_NS . 'icqChatID');

200. }

202. function IMG()
203. {
204. return  new ResResource(FOAF_NS . 'img');

206. }

208. function INTEREST()
209. {
210. return  new ResResource(FOAF_NS . 'interest');

212. }

214. function JABBER_ID()
215. {
216. return  new ResResource(FOAF_NS . 'jabberID');

218. }

220. function KNOWS()
221. {
222. return  new ResResource(FOAF_NS . 'knows');

224. }

226. function LOGO()
227. {
228. return  new ResResource(FOAF_NS . 'logo');

230. }

232. function MADE()
233. {
234. return  new ResResource(FOAF_NS . 'made');

236. }

238. function MAKER()
239. {
240. return  new ResResource(FOAF_NS . 'maker');

242. }

244. function MBOX()
245. {
246. return  new ResResource(FOAF_NS . 'mbox');

248. }

250. function MBOX_SHA1SUM()
251. {
252. return  new ResResource(FOAF_NS . 'mbox_sha1sum');

254. }

256. function MEMBER()
257. {
258. return  new ResResource(FOAF_NS . 'member');

260. }

262. function MEMBERSHIP_CLASS()
263. {
264. return new ResResource(FOAF_NS . 'membershipClass');

266. }

268. function MSN_CHAT_ID()
269. {
270. return  new ResResource(FOAF_NS . 'msnChatID');

272. }

274. function MYERS_BRIGGS()
275. {
276. return  new ResResource(FOAF_NS . 'myersBriggs');

278. }

280. function NAME()
281. {
282. return  new ResResource(FOAF_NS . 'name');

284. }

286. function NICK()
287. {
288. return  new ResResource(FOAF_NS . 'nick');

290. }

292. function PAGE()
293. {
294. return  new ResResource(FOAF_NS . 'page');

296. }

298. function PAST_PROJECT()
299. {
300. return  new ResResource(FOAF_NS . 'pastProject');

302. }

304. function PHONE()
305. {
306. return  new ResResource(FOAF_NS . 'phone');

308. }

310. function PLAN()
311. {
312. return  new ResResource(FOAF_NS . 'plan');

314. }

316. function PRIMARY_TOPIC()
317. {
318. return  new ResResource(FOAF_NS . 'primaryTopic');

320. }

322. function PUBLICATIONS()
323. {
324. return  new ResResource(FOAF_NS . 'publications');

326. }

328. function SCHOOL_HOMEPAGE()
329. {
330. return  new ResResource (FOAF_NS . 'schoolHomepage');

332. }

334. function SHA1()
335. {
336. return  new ResResource (FOAF_NS . 'sha1');

338. }

340. function SURNAME()
341. {
342. return  new ResResource (FOAF_NS . 'surname');

344. }

346. function THEME()
347. {
348. return  new ResResource(FOAF_NS . 'theme');

350. }

352. function THUMBNAIL()
353. {
354. return  new ResResource(FOAF_NS . 'thumbnail');

356. }

358. function TIPJAR()
359. {
360. return  new ResResource(FOAF_NS . 'tipjar');

362. }

364. function TITLE()
365. {
366. return  new ResResource(FOAF_NS . 'title');

368. }

370. function TOPIC()
371. {
372. return  new ResResource(FOAF_NS . 'topic');

374. }

376. function TOPIC_INTEREST()
377. {
378. return  new ResResource(FOAF_NS . 'topic_interest');

380. }

382. function WEBLOG()
383. {
384. return  new ResResource(FOAF_NS . 'weblog');

386. }

388. function WORK_INFO_HOMEPAGE()
389. {
390. return  new ResResource(FOAF_NS . 'workInfoHomepage');

392. }

394. function WORKPLACE_HOMEPAGE()
395. {
396. return  new ResResource(FOAF_NS . 'workplaceHomepage');

398. }

400. function YAHOO_CHAT_ID()
401. {
402. return  new ResResource(FOAF_NS . 'yahooChatID');
403. }
404. }


409. ?>
```

Documentation generated on Fri, 17 Dec 2004 16:14:57 +0100 by phpDocumentor 1.3.0RC3
